# Production of cecropin A antimicrobial peptide in rice seed endosperm

**DOI:** 10.1186/1471-2229-14-102

**Published:** 2014-04-22

**Authors:** Mireia Bundó, Laura Montesinos, Esther Izquierdo, Sonia Campo, Delphine Mieulet, Emmanuel Guiderdoni, Michel Rossignol, Esther Badosa, Emilio Montesinos, Blanca San Segundo, María Coca

**Affiliations:** 1 Centre for Research in Agricultural Genomics (CRAG), CSIC-IRTA-UAB-UB. Edifici CRAG, Campus de la UAB, 08193 Bellaterra, Barcelona, Spain; 2 Institute of Food and Agricultural Technology-CIDSAV-XaRTA, University of Girona, Girona 17071, Spain; 3 Mass Spectrometry Proteomics Platform-MSPP, Laboratoire de Protéomique Fonctionnelle, INRA, Cedex 1, Montpellier 34060, France; 4 CIRAD, Centre de Coopération Internationale en Recherche Agronomique pour le Développement, UMR AGAP, Genetic Improvement and Adaptation of Mediterranean and Tropical Plants, Cedex 5, Montpellier 34398, France

**Keywords:** Rice, Antimicrobial peptides, Cecropin A, Endosperm, Protein bodies, Pathogen resistance, *Fusarium verticillioides*, *Dickeya dadantii*, *Oryza sativa*

## Abstract

**Background:**

Cecropin A is a natural antimicrobial peptide that exhibits rapid, potent and long-lasting lytic activity against a broad spectrum of pathogens, thus having great biotechnological potential. Here, we report a system for producing bioactive cecropin A in rice seeds.

**Results:**

Transgenic rice plants expressing a codon-optimized synthetic *cecropin A* gene drived by an endosperm-specific promoter, either the *glutelin B1* or *glutelin B4* promoter, were generated. The signal peptide sequence from either the *glutelin B1* or the *glutelin B4* were N-terminally fused to the coding sequence of the cecropin A. We also studied whether the presence of the KDEL endoplasmic reticulum retention signal at the C-terminal has an effect on cecropin A subcellular localization and accumulation. The transgenic rice plants showed stable transgene integration and inheritance. We show that cecropin A accumulates in protein storage bodies in the rice endosperm, particularly in type II protein bodies, supporting that the glutelin N-terminal signal peptides play a crucial role in directing the cecropin A to this organelle, independently of being tagged with the KDEL endoplasmic reticulum retention signal. The production of cecropin A in transgenic rice seeds did not affect seed viability or seedling growth. Furthermore, transgenic cecropin A seeds exhibited resistance to infection by fungal and bacterial pathogens (*Fusarium verticillioides* and *Dickeya dadantii*, respectively) indicating that the *in planta*-produced cecropin A is biologically active.

**Conclusions:**

Rice seeds can sustain bioactive cecropin A production and accumulation in protein bodies. The system might benefit the production of this antimicrobial agent for subsequent applications in crop protection and food preservation.

## Background

Antimicrobial peptides (AMPs) are evolutionarily conserved components of the innate immune system of most living organisms. AMPs show a high degree of sequence diversity but share some characteristics including their predominantly cationic character, a high content of hydrophobic residues, and an amphipathic structure. They are also natural antibiotics that exhibit rapid, potent and long-lasting activity against a broad spectrum of pathogens by affecting conserved features of microbial cell membranes [1]. AMPs are emerging as valuable agents for crop protection [2-4], food preservation [5,6], and pharmaceuticals for both human and animal health [7-9] to alleviate the growing problem of conventional antibiotic resistance and the shortage of effective compounds. However, the high cost of chemical synthesis or the low yield obtained via purification from natural sources has limited the use of AMPs in these fields, particularly in applications with little added value.

Plants are promising biofactory systems for AMPs; they are economical to grow, easily scalable and generally regarded as safe because of the low risk of contamination with human and animal pathogens [10]. Plants have successfully been used for the production of different proteins for therapeutic and technological applications [11]. However, little attention has been paid to the use of plants as biofactories for AMPs. Evidence indicating that plants can sustain AMP production can be found in the literature, since plants have been used for the heterologous production of AMPs with the aim of improving host resistance to pathogen infection [3]. No extensive efforts were made in those studies, however, to quantify the amount of AMP produced in the transgenic plants.

Seeds naturally accumulate proteins, packed in a dehydrated optimal biochemical environment for long-term storage. Thus, this organ seems suitable for the production of stable large amounts of AMPs in a compact biomass. In particular, rice seeds are considered as a good biofactory due to high grain yields. Furthermore, rice is easy to transform, can be grown under containment conditions, and the risk of unintended gene flow is minimal compared with other crops [12].

The rice endosperm is an appropriate tissue for the heterologous production of proteins of interest [13-17]. This organ occupies most of the space within the rice seed. Endosperm cells mostly contain starch granules and proteins, and are the major storage protein sink. In rice, 60% to 80% of all storage proteins are glutelins, which are insoluble in neutral saline solutions but soluble in acid or alkaline solutions [18]. They are classified into four groups (*GluA*, *GluB*, *GluC* and *GluD*) based on their amino acid sequence [19]. Some 20% to 30% of all rice seed proteins are alcohol-soluble prolamines [18]. Rice seeds also accumulate a salt-soluble globulin which comprises up to 5% of the seed protein [20]. These storage proteins are densely packed into specialized storage organelles called protein bodies (PBs). There are two types of PBs in rice seeds: PB-I and PB-II. The former are spherical protein inclusions that bud from the endoplasmic reticulum (ER) and in which prolamines are typically accumulated [21,22]. The later, also known as protein storage vacuoles (PSVs), contain glutelin and globulin proteins, and are characterized by their irregular shape, with a diameter of about 2–4 μm, and their highly uniform dense structure. Glutelins aggregate in PB-II and form protein inclusions with crystalline structures [21,23]. PB-II are derived from Golgi or may bypass the Golgi complex [24]. All storage proteins contain an N-terminal signal peptide that mediates translocation into the ER, where the signal peptide is cleaved and the protein is transported to the appropriate storage compartment [25]. The seed storage organelles can contain recombinant proteins, offering stability *in planta* and allowing considerable accumulation [26].

The aim of this study is to explore the feasibility of using rice seed endosperm for the production of AMPs. Cecropin A was chosen as the AMP to be produced, based on its biotechnological potential. Cecropin A is a linear and cationic peptide isolated from insect haemolymph that shows potent lytic activity against important bacterial and fungal phytopathogens [27-29]. Its constitutive accumulation in transgenic rice plants has been shown to confer enhanced pathogen resistance [30]. Previous studies by our group demonstrated that transgenic rice plants constitutively expressing a *cecropin A* gene designed to secret the encoded peptide in the extracellular space had an abnormal phenotype and were not fertile [30]. No effect on plant performance was observed in the transgenic rice plants that accumulated cecropin A in the ER, the plants accumulated low levels of the peptide in their leaves [30]. Here, we report the production and accumulation of bioactive cecropin A in rice endosperm without any impact on seed viability or seedling growth. Two different endosperm-specific promoters were used to drive the expression of a codon-optimized synthetic *cecropin A* gene (*CecA*), namely the *GluB1* and *GluB4* promoters. The N-terminal signal peptide sequence of either the GluB1 or GluB4 protein was fused to the cecropin A sequence. Furthermore, two different *CecA* genes, encoding the cecropin A or cecropin A-KDEL peptide, were assayed to determine the effect of the ER retention signal (KDEL) on peptide accumulation and subcellular localization.

## Results

### Generation of transgenic rice plants for cecropin A production

**Figure 1:**
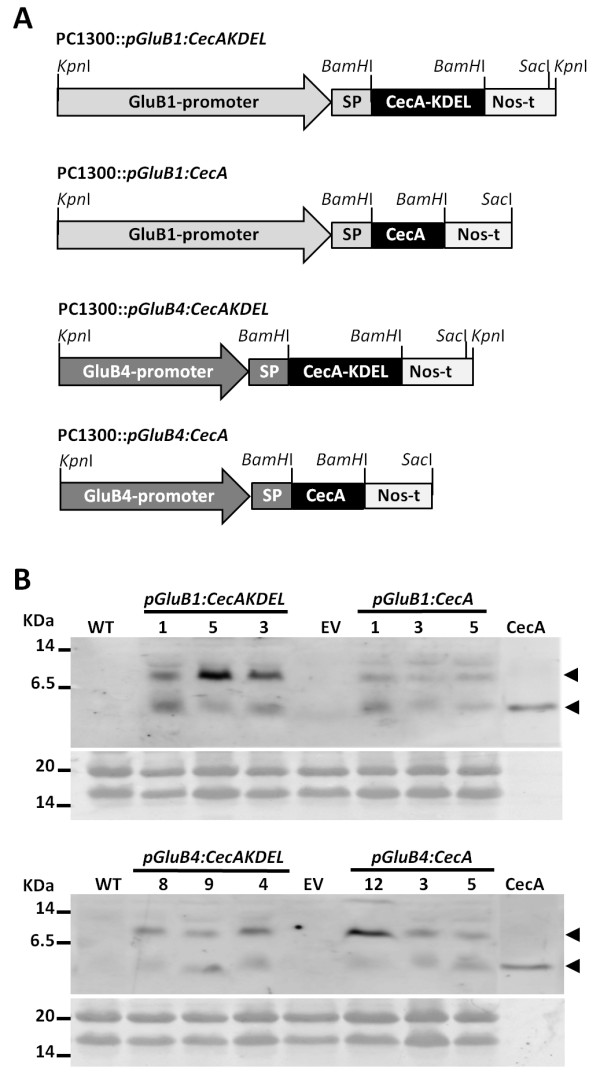
**Generation of transgenic rice plants producing cecropin A. A**. Schematic representation of the constructs. Expression of the synthetic *CecA* genes was controlled by the 2.3 kb *GluB1* or the 1.4 kb *GluB4* promoters and the *nos* terminator. Relevant restriction enzyme sites for cloning purposes are indicated. **B**. Cecropin A accumulation in transgenic rice plants. Immunoblot analysis using anti-cecropin A antibodies of protein extracts from wild-type (WT) or transgenic seeds carrying the empty vector (EV) or the transgenes indicated. For comparative purposes, 50 ng of synthetic cecropin A was run simultaneously in the tricine-SDS gels. Lower panels show Ponceau staining of protein samples.

Four different constructs were prepared for the expression of synthetic codon-optimized *CecA* genes in rice seeds; they are shown in Figure 1A. They contain the promoter of either the *GluB1* or the *GluB4* gene, which encode the major rice seed storage proteins, to drive seed-specific expression of the synthetic genes. An endosperm-specific expression pattern has been reported for these two promoters [31]. Each construct incorporates a different chimeric *CecA* gene designed to study different targeting mechanisms of the encoded peptide. The chimeric genes consist of the N-terminal signal peptide sequence of the corresponding glutelin B1 or B4 protein fused to the coding sequence of the cecropin A peptide; two of them also include the sequence encoding the C-terminal ER retention signal (KDEL).

Transgenic rice plants were produced by *Agrobacterium*-mediated transformation using the hygromycin resistance gene as the selectable marker. After each transformation, hygromycin-resistant plants were obtained and transgene integration was verified by PCR analysis of (Additional file 1). No apparent adverse effects on the plant phenotype were observed under greenhouse conditions. Six independent lines per transformation event were selected to obtain the T2 homozygous progeny plants. The stability of the transgene integration and inheritance was monitored across generations by the hygromycin resistance phenotype encoded in the T-DNA.

### Accumulation of cecropin A in rice seeds

**Figure 2:**
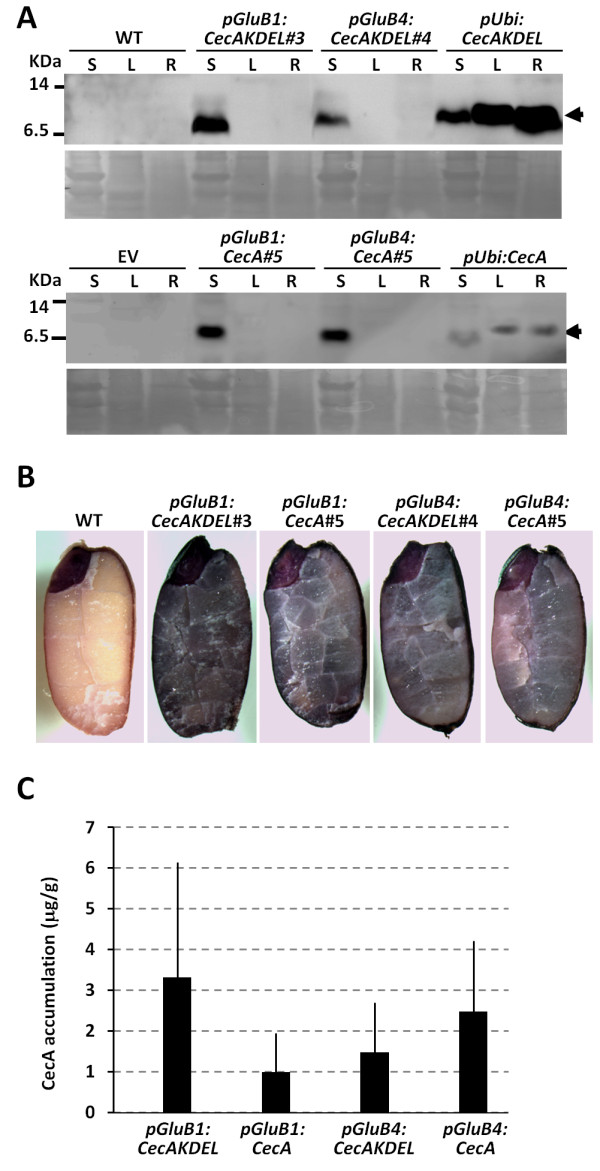
**Cecropin A accumulates in seeds but not in vegetative tissues of rice plants. A**. Immunoblot analysis of cecropin A in protein extracts (15 μg) from roots (R), leaves (L) and seeds (S) of wild-type (WT) and transgenic plants carrying the empty vector (EV) or the transgenes indicated. Lower panels show Ponceau staining of protein samples. **B**. *In situ* immunolocalization of cecropin A in WT and transgenic mature rice seeds. Representative images for each transformation event are shown (6 seeds per line, 3 lines per event and 2 independent assays). **C**. Cecropin A accumulation as estimated by immunoblot analysis of protein extracts from transgenic mature seeds in T3 generation compared to known amounts of synthetic cecropin A. The values correspond to the mean of estimated amounts of cecropin A per seed weight of at least three independent transgenic lines per transformation event and the bars to standard deviation. Results represent 3 independent experimental replicas.

To determine whether the transgenic lines accumulated the transgene product, the presence of cecropin A in seed protein extracts was analysed by immunoblot assays using specific antibodies. As shown in Figure 1B, the anti-cecropin A antibodies reacted with two major bands in the protein extracts obtained from transgenic seeds, which were absent in the extracts obtained either from wild-type or from the empty vector transformed seeds. The strongest signal corresponded to a peptide with a mobility around 8 kDa, which is compatible with a cecropin A dimer conformation (the theoretical molecular weight of cecropin A is 4 kDa), as reported previously for lines constitutively producing cecropin A [30] and as also shown in Figure 2A. A band with an estimated mobility approximately 4 kDa that migrates similarly to the synthetic cecropin A was also detected in the cecropin A lines, which could correspond to the cecropin A monomeric peptide. The cecropin A dimer was consistently detected in all the transgenic lines and in all the immunoblot analyses conducted during the course of this work; whereas the monomeric cecropin A was not always detected, suggesting that the dimeric conformation was more stable. Figure 1B also shows that cecropin A peptides were detected in all the lines carrying any of the four chimeric genes, indicating that the *CecA* transgenes were properly expressed and their corresponding products accumulated in the rice seeds.

We then examined whether the *glutelin* promoters specifically direct *CecA* gene expression specifically to seed and not to vegetative tissue, such as roots or leaves. To this end, protein extracts were prepared from the roots and leaves of representative lines per each transgene and subjected to Western blot analysis. As positive controls, protein extracts from transgenic lines expressing *CecA* genes controlled by the maize *ubiquitin-1* promoter were also included in this analysis. As shown in Figure 2A, cecropin A was detected in the protein extracts of seeds carrying any of the *CecA* genes controlled by *glutelin* promoters, whereas they were absent in the extracts from leaves and roots of the same plants. As expected, positive reactions were detected in the leaf and root protein extracts of the lines constitutively expressing *CecA* genes. Thus, this analysis confirmed that cecropin A accumulated in rice seeds, but not in the vegetative tissues, when expression was controlled by either the *GluB1* or the *GluB4* promoter.

To further characterize the cecropin A lines, the distribution of the heterologous peptide in the seeds was analysed by *in situ* immunodetection. As shown in Figure 2B, a positive reaction was detected in the mature seeds of transgenic plants harbouring any of the four transgenes. Specific immunological reactions were detected in the endosperm of cecropin A seeds as compared to wild-type seeds. An unspecific reaction in embryo tissue was also observed in all the seeds analysed, including wild-type and empty vector seeds (Figure 2B). Therefore, the transgenic lines produced and accumulated cecropin A in the rice seed endosperm.

A comparative analysis of cecropin A accumulation in the different transgenic lines generated in this work was carried out. By comparing band intensities with those of known amounts of synthetic cecropin A, the cecropin A content in the lines was determined (Figure 2C). This study revealed variability in cecropin A accumulation between transgenic lines harbouring the same transgene, as well as in lines harbouring different transgenes. The accumulation levels ranged from 0.5 to 6 μg/g seed tissue. Similar accumulation levels were observed using either the *GluB1* or *GluB4* promoter, suggesting that the observed variability is associated with transgenesis rather with the activity of one or another promoter. Furthermore, the cecropin A levels were similar in seeds harbouring the transgenes with or without the KDEL extension sequence, indicating that the KDEL signal did not enhance accumulation of the peptide. Only one line carrying the *pGluB1*:*CecAKDEL* transgene (line 5) appeared to accumulate higher levels than the other transgenic lines. In addition, the observed variability in accumulation did not correlate with the transgene copy number of each line, as most of the lines generated contained one or two transgene insertions as estimated by quantitative PCR (Additional file 2).

### Cecropin A accumulates in PB-II

**Figure 3:**
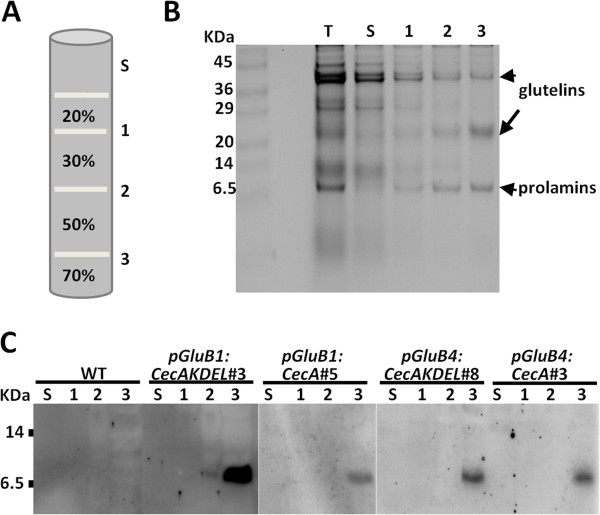
**Cecropin A accumulates in dense subcellular fractions. A**. Diagram of the sucrose (w/v) gradient steps. The total homogenate (T), supernatant (S) and the interfaces (1, 2, and 3) are indicated. **B**. Protein profiles of equivalent amounts of the recovered gradient fractions, separated in SDS gels and Coomassie-blue stained. **C**. Immunoblot analysis of equivalent amounts of gradient fractions isolated from untransformed (WT) and transgenic seeds carrying the genes indicated separated on tricine-SDS-gels and detected with anti-cecropin A antibodies.

Cecropin peptides are known to be highly susceptible to plant proteases, which limits their accumulation in plant tissues [27,32,33]. A priori, directing the cecropin A to endosperm PBs would protect it from host proteases. In order to determine whether cecropin A were accumulated in PBs of rice seeds, subcellular fractionation by centrifugation on a sucrose gradient was performed and the fractions were subjected to immunoblot analysis. Coomassie blue staining of equivalent protein fractions showed a clear enrichment of glutelin and prolamine proteins in the densest fraction (fraction 3, Figure 3A-B). Immunoblot analysis of gradient fraction proteins revealed the presence of cecropin A in fraction 3 in all the lines, whether they contained the KDEL extension or not (Figure 3C). These results demonstrate that cecropin A accumulates in dense seed protein organelles.

**Figure 4:**
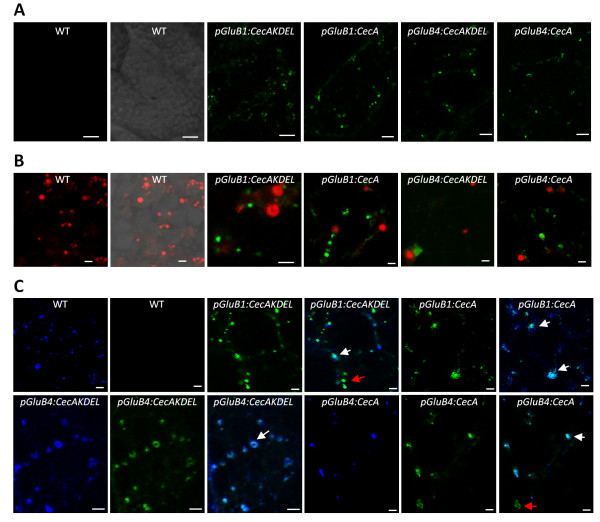
**Cecropin A accumulates in type II protein bodies.** Confocal fluorescent images of peripheral endosperm cells from wild-type (WT) or transgenic seeds carrying the transgenes indicated. Cecropin A was immunodetected and visualized in green using an AlexaFluor488-conjugated secondary antibody. Prolamine-containing PBs (PB-I marker) were stained with rhodamine B hexyl ester and visualized in red. Glutelin-containing PBs (PB-II marker) were immunodetected using AlexaFluor647-conjugated anti-glutelin antibodies and visualized in blue. Sections were single fluorescent labelled for cecropin A **(A)** or double fluorescent labelled for cecropin A and prolamines **(B)** or for cecropin A and glutelins **(C)**. Images correspond to sequential scan single slides. Merged images are shown in **B** and **C**. Scale bars correspond to 10 μm **(A)** or 2 μm **(B** and **C)**. Red arrows indicate single immunolabelled vesicles and white arrows double immunolabelled vesicles.

Furthermore, the subcellular localization of cecropin A was analysed by immunofluorescence microscopy in rice seed sections. As shown in Figure 4A, cecropin A (labelled in green) accumulated in small cytoplasmic vesicles of peripheral endosperm cells but it did not in wild-type seeds. These vesicles showed a similar appearance and abundance in all the cecropin A lines, as well as in the cecropin A-KDEL lines. As shown, they are smaller and less spherical than the rhodamine B-stained vesicles (Figure 4B, labelled in red), corresponding to prolamine-containing PBs (PB-I). Rhodamine B hexyl ester binds strongly to rice prolamine and it is regularly used for fluorescent labelling of PB-I [34]. The merged images in Figure 4B clearly show that cecropin A was absent from PB-I, since no colocalization is observed. When the glutelin-containing PBs (PB-II) were immunolabelled, fluorescence was detected in bodies that have a similar shape and size to bodies containing cecropin A (Figure 4C, labelled in blue). The merged images show frequent colocalization of glutelin and cecropin A signals; while some bodies were stained only in green or blue. These results indicate that cecropin A, as well as the KDEL-tagged cecropin A, were packed into PB-II together with glutelin storage proteins in the endosperm cells of transgenic rice seeds. Therefore, the same subcellular localization of cecropin A in PB-II is observed when either the GluB1 or the GluB4 signal peptides is added at the N-terminal, and independently of the KDEL signal at the C-terminal.

### *Identification of* in planta-*produced cecropin A by MS analysis*

**Figure 5:**
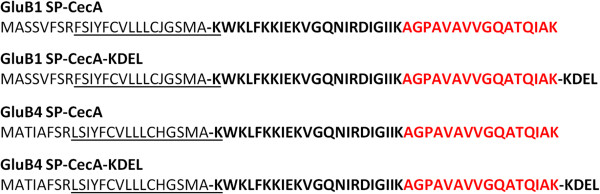
**Amino acid sequence of chimeric cecropin A peptides.** Plain text sequences correspond to the signal peptide sequence of glutelin B1 (GluB1 SP) or glutelin B4 (GluB4 SP) storage proteins; bold sequences to the mature cecropin A peptides; red sequences to the tryptic peptide identified by MS analysis, and underlined sequences to tryptic peptides undetected by MS analysis.

To verify that *in planta*-produced cecropin A was correctly synthesized, subcellular fractions enriched in PBs were analysed by mass spectrometry (MS). To do this, protein fractions were separated by SDS-PAGE. The gel pieces in the molecular weight range for immunoreactive cecropin A were then cut, digested with trypsin, and MS analysed. The peptide AGPAVAVVGQATQIAK (monoisotopic mass 1479.84 Da), corresponding to the C-terminal region of the cecropin A sequence (Figure 5), was unequivocally identified in all the transgenic samples and was not detected in wild-type samples. This peptide was identified in the gel slices in which monomeric and dimeric cecropin A forms were immunodetected (4 and 8 kDa, respectively). The expected tryptic peptides FSIYFCVLLLCHGSMAK and LSIYFCVLLLCHGSMAK for the GluB1 or GluB4 signal peptide-cecropin A combinations were not detected by MS analysis in any of the transgenic samples, this suggests that the peptide was correctly processed in each case. These MS data confirmed the presence of cecropin A in the transgenic rice seeds.

### Resistance of cecropin A rice seeds to seed-borne pathogens

**Figure 6:**
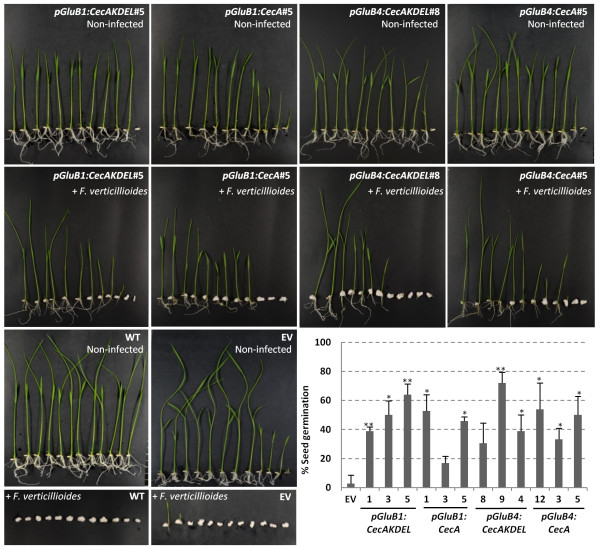
**Resistance of cecropin A rice seeds to *Fusarium verticillioides*.** Phenotypical appearance of wild-type (WT) and transgenic seedlings carrying the transgenes indicated or the empty vector (EV) 7 days after germination in control conditions (non-infected) or inoculated with *F. verticilliodes* spore suspensions (10^5^ spores/ml). Pictures are representative of 3 independent lines per transformation event and at least 3 independent assays. The graph shows the germination rate of transgenic seeds inoculated with *F. verticillioides* referred to wild-type seeds. The values correspond to the mean of three independent assays and the bars to standard errors. Asterisks denote statistically significant differences with wild-type and empty vector plants (*p < 0.05, **p < 0.01, ANOVA).

To test the functionality of the cecropin A produced in the rice seeds, transgenic seeds were evaluated for resistance to rice seed pathogens; both fungal and bacterial. Initially, cecropin A-seeds were inoculated with spores of *Fusarium verticillioides*, a fungal pathogen of rice seeds. Cecropin A-seeds were able to germinate and survive in the presence of this fungal pathogen. However, under the same experimental conditions, both wild-type and empty vector transformed seeds failed to germinate (Figure 6). The quantification of the germination rate after fungal infection revealed a higher germination capacity of cecropin A-seeds than that of wild-type or empty vector seeds (Figure 6). Some differences in the resistance to infection by *F. verticillioides* between the lines were observed. Disease resistance correlated with cecropin A accumulation, the seeds accumulating lower cecropin A levels exhibited lower germination capacity in the presence of the fungal pathogen. For instance, line *pGluB1:CecA*#3 showed low cecropin A accumulation (Figure 1B) and low germination rates after inoculation with *F. verticillioides* (Figure 6).

**Figure 7:**
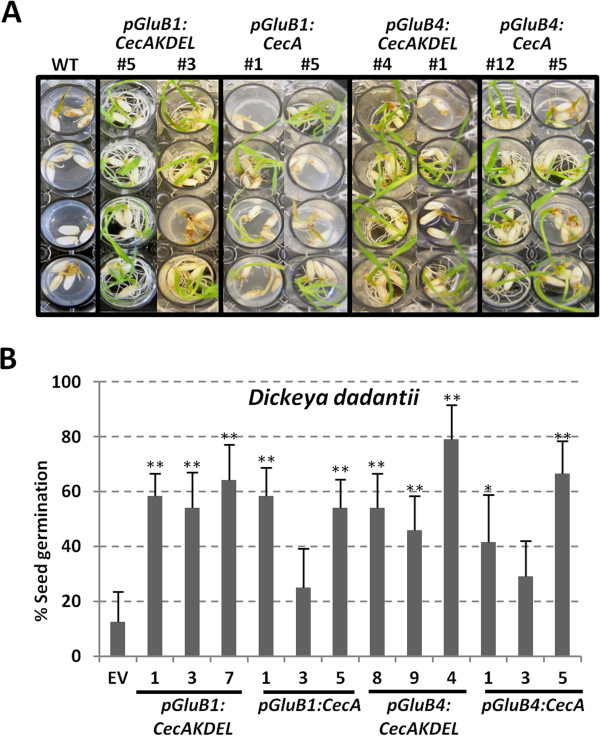
**Resistance of cecropin A rice seeds to *Dickeya dadantii*. A**. Phenotypical appearance of wild-type (WT) or transgenic seedlings carrying the transgenes indicated after 7 days of germination in contact with *D. dadantii* bacterial suspensions (10^4^ CFU/ml). Pictures are representative of at least 2 independent assays. **B**. Germination rate of transgenic seeds inoculated with *D. dadantii* referred to wild-type seeds. The values correspond to the mean of two independent assays and the bars to standard errors. Asterisks denote statistically significant differences with wild-type and empty vector plants (*p < 0.05, **p < 0.01, ANOVA).

Finally, cecropin A-accumulating seeds were assayed for resistance against *Dickeya dadantii*, a bacterial pathogen of rice. Germination of wild-type and empty vector seeds in bacterial suspensions was severely affected, whereas cecropin A-accumulating seeds were able to germinate and grow under the same experimental conditions (Figure 7A). The quantification of the cecropin A-seed germination rates after seven days in contact with the bacterial pathogen and in comparison with wild-type seeds, showed a clear improvement in seed germination (Figure 7B). All the seeds assayed, transgenic and wild-type, showed the same germinative capacity in the absence of bacterial infection (data not shown). These results demonstrate that cecropin A accumulation in rice seeds confers protection against seed fungal and bacterial pathogens, suggesting that the *in planta*-produced cecropin A is biologically active.

## Discussion

In the present study, we generated transgenic rice plants that produce bioactive cecropin A in their seed endosperm. Stable integration and inheritance of transgenes was demonstrated. Our transgenic rice seeds tolerated the accumulation of this bioactive peptide without any major change in germination rate or seed viability; thus indicating that rice seeds can sustain the production of this AMP. Therefore, our work demonstrates the usefulness of the *GluB1* and *GluB4* promoters to drive strong and tissue-specific expression of *CecA* genes in rice seeds. High activity in the rice endosperm was reported for these two promoters, with *GluB4* activity being slightly higher than that of *GluB1*[31]. Consistently with those findings, we detected specific accumulation of cecropin A on seed endosperms, and not in vegetative tissues, when *CecA* gene expression was driven by either of these two promoters. While, similar accumulation levels were observed with the two promoters assayed, the accumulation levels were higher than those of seeds of transgenic plants that constitutively express the *CecA* gene controlled by the strong maize *ubiquitin-1* promoter. Moreover, the endosperm-specific expression of *CecA* genes had no negative effects on the normal growth and development of the rice plant; whereas important effects on plant fitness have been reported in transgenic rice plants that constitutively express transgenes encoding cecropin A or cecropin A-derived AMPs [30,35]. As an additional benefit, the seed-specific accumulation of the cecropin A limits the exposure of the beneficial microbes in the rhizosphere to AMPs.

Concerning the study of targeting mechanisms, in this work we analysed the effects of the N-terminal signal peptide of a rice glutelin, either GluB1 or GluB4, and of the presence or absence of C-terminal KDEL tag. These signal peptides are known to guide translocation of the GluB1 and GluB4 storage proteins into the ER lumen and to be essential for transfer to PBs [25]. Immunofluorescence and microscopic visualization of the transgenic seed tissues revealed that cecropin A accumulated in the glutelin-containing storage vacuole of endosperm cells, when either signal peptide was fused to the cecropin A. This subcellular localization was observed in all transgenic seeds, including those that accumulated the cecropin A tagged with the KDEL signal. Thus, it appears that the GluB1 or GluB4 N-terminal signal peptide includes the sorting signals necessary to direct this peptide to the PB-II and overpowers the KDEL ER retention signal. Unexpected accumulation of KDEL-tagged recombinant seed proteins has previously been reported and is not fully understood [36-39]. Most KDEL-tagged recombinant seed proteins have been reported to accumulate primarily, or exclusively in ER-derived PBs or PB-I [39]. Presumably, protein sorting to PBs might not only rely on the presence of specific targeting signals in the polypeptide but also might depend on the intrinsic properties of the protein or possible interactions with other proteins in the ER lumen [24,39]. For instance, protein aggregation has been reported as a determinant of sorting to PBs [24,40]. There is then the possibility that the specific physico-chemical properties of the cecropin A may be responsible for its localization in the PB-II. In favour of this hypothesis, the cecropin A peptide has amphipathic properties with a structure where hydrophobic residues are clustered into a separated domain, which may confer a tendency for self-aggregation, as proved by its dimeric form in the rice protein extracts. Moreover, cecropin A is a strongly cationic peptide and this may facilitate its interaction with the acidic glutelins during their transport to PSVs, and then they may sort together to the same organelle. However, some cecropin A-containing bodies from which the glutelin proteins were excluded were also visualized, indicating that cecropin A could also accumulate in other ER-derived PBs. Further studies are needed to clarify the molecular determinants of cecropin A accumulation into the PB-II in seeds. Be that as it may, the important result is the compartmentalization of the cecropin A antimicrobial peptide inside subcellular organelles, which protect the peptide from endogenous proteases, thereby offering stability and allowing it to accumulate in rice tissues. Cecropin peptides are known to be highly susceptible to plant proteases and therefore their accumulation in plant tissues is not straightforward [32]. In addition, sequestration of this AMP into a storage organelle, such as PBs, would reduce its toxicity to the host plant and cecropin A seeds were indeed viable and showed a normal germination rate.

Transgenic plants expressing the *cecropin A* gene under the control of either promoter with the KDEL extension, or not, were evaluated in terms of cecropin A accumulation in seeds. Accumulation ranged from 10 to 100 ng per seed, as measured via immunoblot analysis. There were differences in cecropin A accumulation, which were mainly associated with variability of transgene expression between independently generated lines for each transformation event. Even the attachment of the KDEL signal to cecropin A appears not to have an important effect on its accumulation in rice seeds (i.e., the *pGluB1:CecAKDEL* and *pGluB1:CecA*, or *pGluB4:CecAKDEL* and *pGluB4:CecA* lines accumulated similar levels of cecropin A. This is despite the fact that several reports indicate that the presence of the KDEL signal sequence enhances the stability and yield of some recombinant proteins in seeds [14,41]. Rice endosperm has been used to produce several valuable recombinant proteins, including antibodies, vaccines and other pharmaceuticals [13-15,39,41-43]. For several of these proteins, the reported accumulation was higher than the cecropin A levels in our transgenic seeds; ranging from 1 μg/seed for the human interleukin-10 protein [44], to the 60 μg/seed for the chimeric tolerogen 7Crp protein [41], and up to 200 μg/seed for chimeric tolerogen TPC7 [39]. However, difficulties in accumulating small peptides (of fewer than 50 residues) in rice endosperm have also been reported and associated with transgene silencing and instability of transgene products in plant cells [45-48]. Thus small peptides have generally been produced through tandem repeats or fusion to carrier proteins [41,45-49], requiring downstream processing to deliver the peptide of interest. Taking into account the peptide size, the amount of the small cecropin A peptide (37 amino acids, 4 kDa) accumulated in rice seeds ranged from 2.5 to 25 pmols/seed. These values are within the average to the reported accumulation of peptides when considering only the size of the peptide with the total fusion protein, such as 1.18 or 51.47 pmols/seed of the chimeric tolerogen 3Crp [47,48] or 6.25 pmols/seed of the tolerogen Cryj I and Cryj II [50]. Additionally, underestimation of cecropin A accumulation based on immunoblot detection cannot be ruled out, since difficulties in Western blot analysis and immunodetection of other basic short peptides have been reported [51]. Nevertheless, the usefulness of producing single cecropin A peptides is not only determined by the level of accumulation in transgenic rice seeds, but also by the potency of cecropin A as an antimicrobial agent for the target pathogens without additional processing. In this sense, previous studies on the antimicrobial activity of cecropin A and cecropin A-derived peptides have shown their effectiveness at very low micromolar concentrations in terms of growth inhibition of economically important phytopathogens, including the fungi *F. solani*, *F. verticillioides* and *Phytophthora infenstans*, and the bacteria *Erwinia amylovora, Pseudomonas syringae* and *Xanthomonas axonapodis*[27,28,52,53].

The targeting of endosperm PBs not only conferred stability and reduced the toxicity of cecropin A, thereby allowing its accumulation in plant cells, but it also facilitated its purification from rice seeds. PBs are dense organelles that can easily be isolated by centrifugation [26,54]. This is particularly relevant here, since downstream processing of plant material to purify products greatly increases the production costs of recombinant proteins when using plants as biofactories [10]. A simple procedure based on two-step centrifugation was implemented here to obtain enriched cecropin A-containing PB fractions. Further studies are required to set up and optimize large-scale purification of cecropin A from seeds for potential applications in crop protection or food preservation. The production system for cecropin A described here using rice endosperm as biofactories can potentially be extended to other AMPs, although this needs to be evaluated for AMPs of different sizes, structures or mechanisms of action.

Finally, the observations that the *in planta*-produced cecropin A is biologically active and that accumulation of cecropin A in rice seeds confers protection against fungal and bacterial pathogens have additional implications for plant protection. In the present work, disease resistance against two important rice pathogens was evaluated. The fungus *F. verticillioides* has been associated with the bakanae disease in rice [55], which occurs widely throughout Asia and sporadically in other rice producing areas, and causes important crop losses worldwide. Moreover, *F. verticillioides* is a seed-borne and seed-transmitted pathogen that not only causes yield losses but also decreases the quality of grain by producing hazardous mycotoxins [56]. The cecropin A-seeds also showed enhanced resistance against *D. dadantii,* previously known as *Erwinia chrysanthemi*, the causal agent of foot rot of rice [57,58]. Resistance to *F. verticillioides* and *D. dadantii* in transgenic seeds indicates that cecropin A accumulation could be a useful strategy for engineering broad-spectrum protection in rice grain.

## Conclusions

Rice seeds can sustain the production of biologically active cecropin A, and presumably other antimicrobial peptides with similar properties. Confining the accumulation of AMP within subcellular compartments, specifically protein bodies, and limiting its production to the rice endosperm avoids the potential negative impact of its production in seed viability and seedling growth. This work has implications for molecular farming since it demonstrates the potential of rice seeds as biofactories for antimicrobial peptides and also for plant protection by showing that production of the antimicrobial peptide cecropin A is a useful strategy for engineering broad-spectrum protection against pathogen infection in rice grains.

## Methods

### Isolation of rice glutelin gene promoters

The promoters and the signal peptide sequence of the two rice *glutelin* genes, *GluB1* and *GluB4,* were amplified by PCR from rice (*Oryza sativa* cv. Senia) genomic DNA using Taq DNA polymerase (Invitrogen). The primer pairs used for the amplification of each promoter are indicated in Additional file 3. The amplified fragments were inserted into the pGEMT-easy vector (Promega) and their nucleotide sequences determined.

### Construction of plant expression vectors

Four vectors containing a codon-optimized synthetic *CecA* gene were prepared for plant transformation. The scheme of the constructs is presented in Figure 1A. Two of them were designed for the production of cecropin A and the other two for C-terminal KDEL-tagged cecropin A. *CecA* gene expression was under the control of the endosperm-specific promoters of either the *GluB1* or the *GluB4* genes including the signal peptide sequence for the corresponding glutelin protein, and the terminator signal of the *nopaline synthase* gene. For vector preparation, the *nos* terminator was inserted as a *Bam*HI-*Sac*I fragment downstream of the promoter fragments in the pGEMT-easy vector; these restriction sites were incorporated into the oligonucleotides used for the PCR amplification of the *nos* terminator. Next, the synthetic *cecA* genes were inserted as *BamH*I fragments between the promoter and the *nos* terminator. The corresponding fragments were obtained by PCR amplification using the oligonucleotides that incorporated the *BamH*I restriction sites (Additional file 3) from the previously described synthetic *CecA* genes [30]. Finally, the complete cassettes for the expression of the *CecA* gene under the control of the *glutelin* promoters as *Kpn*I-*Sac*I fragments, and for the expression of the *CecA-KDEL* gene as *Kpn*I fragments, were cloned into the pCAMBIA 1300 vector. This resulted in the plasmids presented in Figure 1A. All the constructs used for rice transformation were verified by nucleotide sequencing.

### Production of transgenic rice plants

Transgenic rice lines (*O. sativa* cv. Senia or cv. Ariete) were produced by *Agrobacterium*-mediated transformation of embryonic callus derived from mature embryos, as described previously [59]. The expression vector constructs were transferred to *Agrobacterium tumefaciens* EHA105 [60]. The parent pCAMBIA 1300 vector already contains the *hygromycin phosphotransferase* gene (*hptII*) in the T-DNA region, affording hygromycin resistance. Transgene insertion was confirmed in the regenerated plants by PCR analysis using leaf genomic DNA as the template. The positive transformants were grown under containment greenhouse conditions to obtain homozygous transgenic lines in the T2 generation. Homozygous lines were identified by segregation of hygromycin resistance. The transgene copy number was estimated using quantitative PCR in the T2 homozygous lines by comparison with standard curves for the *CecA* gene at different DNA concentrations (Additional file 2), using a previously described method [35,61]. Rice plants transformed with the empty vector (pCAMBIA 1300) were also produced for this study; rice plants constitutively expressing the *CecA* gene were also assayed [30]. All rice plants were grown at 28°C ± 2°C under a 14 h/10 h light/dark photoperiod.

### In situ immunodetection of cecropin A in whole seeds

Cecropin A accumulation in the transgenic rice seeds was analysed by *in situ* immunodetection using antibodies against cecropin A [30], according to the method of Qu and collaborators [62] with minor modifications. These included a stronger and extended blocking procedure (overnight incubation of sections in 10% skimmed milk in TBST), reaction with cecropin A antiserum (2 hours incubation with a 1:500 dilution), and the colorimetric detection of the antigen-antibody complexes using the NBT/BCIP substrate of alkaline phosphatase (Roche).

### Immunolocalization of cecropin A in PBs

Dehulled mature seeds were used for immunofluorescent detection of cecropin A in rice PBs using 10% skimmed milk as a blocking reagent [63]. Immunoreaction with rabbit anti-cecropin A antibodies (1:200 dilution) were visualized using the fluorescent labelled AlexaFluor488 anti-rabbit IgG as secondary antibodies (Molecular Probes, 1:5000 dilution). Anti-glutelin antibodies (kindly provided by Dr. Okita, Washington State University, USA) were conjugated to the fluorophore AlexaFluor647 using the APEX antibody labelling kit (Molecular Probes) and were used for fluorescent immunodetection of PB-II. PB-I were fluorescently labelled with rhodamine B hexyl ester (Molecular Probes). Fluorescent endosperm cells were analysed with a confocal laser scanning microscope (Leica TCS-SP5II). The AlexaFluor488 fluorophore was excited with a blue argon ion laser (488 nm) and the emitted light was collected between 500 and 550 nm. The rhodamine was excited with a HeNe laser (543 nm) and the emitted light was collected between 570 and 650 nm; and the AlexaFluor647 fluorophore was excited with a HeNe laser (633 nm) and the emitted light was collected between 650 and 750 nm. The resulting images were processed using Leica LAS-AF software (version 1.8.2).

### Subcellular fractionation

Ten dehulled mature seeds per line were water imbibited for one hour, and then ground in a mortar at 0°C in 5 ml of homogenization buffer (HB, 10 mM Tris–HCl pH 7.5, KCl 50 mM, MgCl2 10 mM, EDTA 10 mM and plant protease inhibitors) containing 10% sucrose. The homogenates were filtered through two layers of Miracloth (Calbiochem) to remove tissue debris and centrifugated at 100 × g for 5 min at 4°C. Aliquots of the supernatants (3 ml) were layered onto discontinuous sucrose density gradients (20%, 30%, 50%, and 70% w/v) in HB buffer and centrifugated at 4°C for 2 h at 24000 × g in a Beckman SW40 Ti rotor. Equivalent aliquots of supernatant, interphase fractions and pellet were analysed by SDS-PAGE followed by protein staining in Coomassie blue or immunoblot using specific antibodies.

### Preparation of protein extracts and immunoblot analysis

Protein extracts were prepared from dehulled mature seeds (10 seeds, 200 mg), after one hour water imbibition, using a simplified method for enrichment in dense organelles. The same protocol was used for vegetative tissues (200 mg). The plant material was ground and homogenized in a sucrose-containing extraction buffer (10 mM phosphate buffer pH7.5, 0.6 M sucrose). Then the cellular debris and starch were removed from the homogenates by low speed centrifugation (200 x g), and PB enriched fractions were obtained by high speed centrifugation (2000 × g) and resuspended directly in SDS-loading buffer. Protein extracts were separated on tricine-SDS-PAGE (16.5%), transferred to a nitrocellulose membrane (Protran 0.2 μm) and immunodetected as described previously [30]. To determine cecropin A accumulation in seed protein extracts, different amounts of cecropin A (GeneScript) were used as standards. Chemiluminescent reaction was captured with an *ImageQuant*™ *LAS4000* (GE Healthcare) digital imaging system. Signal intensity was quantified using *Multi*-*Gauge* V3.0 (FujiFilm) software. Quantification was performed in 3 independent experimental replicas using at least 3 independent lines per transgene and 10 seeds per line.

### Mass Spectrometry Analysis

Subcellular fraction samples containing PBs were diluted in 5 volumes of ultrapure water and precipitated by centrifugation for 45 min at 75000 × g. Then, proteins were resuspended in SDS-loading buffer and separated by tricine-SDS-PAGE (16.5%). Two bands were excised from the gel between the dye front and the 20 kDa marker (Sigma). For in-gel digestion, gel pieces were washed in 25 mM NH_4_HCO_3_, pH8, 50% acetonitrile, dehydrated with 100% acetonitrile and digested with 50 μl of a trypsin (Promega) solution in 25 mM NH_4_HCO_3_, pH8 overnight at 37°C. Peptides were extracted successively with 2% v/v formic acid and acetonitrile/water (80/20 v/v). Extracts were combined, dried and dissolved in 0.1% formic acid before LC-MS analysis.

The protein digests were analysed using a QTOF mass spectrometer (Maxis Impact; Bruker Daltonik GmbH), interfaced with a nano-HPLC Ultimate 3000 (Dionex). Samples were first loaded onto the pre-column (C18 PepMap100, 300 μm × 5 mm, 5 μm, 100 A, Dionex) at a flow rate of 20 μl/min for 5 minutes with solvent A (0.1% formic acid, 2% acetonitrile in water, v/v/v). After pre-concentration, peptides were separated in the reversed-phase column (C18 PepMap100, 75 μm × 250 mm, 3 μm, 100 A, Dionex) at a flow rate of 0.3 μl/min using a two-step linear gradient from 7% to 25% solvent B (0.1% formic acid, 90% acetonitrile in water, v/v/v) from 0 to 70 min and from 25% to 40% solvent B, from 70 to 90 min, and eluted into the mass spectrometer. The instrument was operated in the positive ion mode and the captive-spray source parameters were: a capillary voltage of 1300 V, a dry gas flow rate of 4 l/min at 150°C. After an initial MS scan at 5 Hz over the mass range of 50–2200 Th, the 30 most intense precursors were fragmented by collision-induced dissociation. The MS/MS raw data were analysed using Data Analysis software (Bruker Daltonik GmbH) to generate the peak lists. A local database including the cecropin A sequence was queried using the Mascot search engine (v. 2.2.04; Matrix Science) with the following parameters: trypsin as enzyme, 1 missed cleavage allowed, oxidation of methionine as variable modification, 15 ppm in MS and 0.05 Da in MS/MS.

### Disease resistance assays

Resistance to *F. verticillioides* was assayed in transgenic rice seeds by inoculation with spore suspensions at different concentrations (10^3^ or 10^5^ spores/ml) on MS medium without sucrose as previously described [64]. Inhibition of germination was determined by comparison to the seeds inoculated with sterile water 7 days post imbibition. The *F. verticillioides* isolate used in this work was collected from rice plants in Spain and provided by the Plant Protection Facilities of the Generalitat de Catalunya.

Resistance of cecropin A-seeds to *D. dadantii* (formely known as *Erwinia chrysanthemi* isolate AC4150) was evaluated by implementing a high-throughput assay derived from a previously described assay [64]. The seeds were germinated in 24-well plates (3 seeds per well) in 1 ml of sterile water or bacterial culture suspensions (10^4^ CFU) for 7 days. Inhibition of germination was evaluated as the ratio to non-inoculated seeds and by comparison to wild-type untransformed seeds.

## Competing interest

The authors declare no competing interests.

## Authors’ contribution

MB carried out the rice transformation experiments, molecular characterization and phenotype analysis of the transgenic lines, including the fungal infection assays. LM prepared all the constructs for rice transformation and contributed to the rice transformation as well as the molecular and phenotype analysis of transgenic lines, including the bacterial infection assays. EI and MR carried out the MS analysis. SC participated in the design of the study and helped to write the manuscript. DM and EG contributed to the rice transformation experiments. EB participated in the bacterial infection assays. EM, BSS and MC conceived, designed and coordinated the study. MC wrote the manuscript. All the authors read and approved the final manuscript.

## Supplementary Material

Additional file 1 **Confirmation of the transgene insertion in the genome of transgenic rice plants.** PCR analysis on genomic DNA purified from leaves of wild-type (wt) or transgenic lines carrying the indicated transgenes. Plasmidic DNA was used as a positive control (+). Arrows indicate the position of the specific oligonucleotides used for PCR amplification. The size of amplified fragments showed full length transgene insertion. Click here for file

Additional file 2 Estimated transgene copy number in the transgenic lines by qPCR analysis. Click here for file

Additional file 3 **List of primers used in the study.** Underlined are the restriction sites used for cloning purposes. Click here for file
