# Supplementary figures and images for: Production of cecropin A antimicrobial peptide in rice seed endosperm
Source: BMC Plant Biol. 2014 Apr 22;14:102. doi: 10.1186/1471-2229-14-102 (PMC4032361; doi:10.1186/1471-2229-14-102)

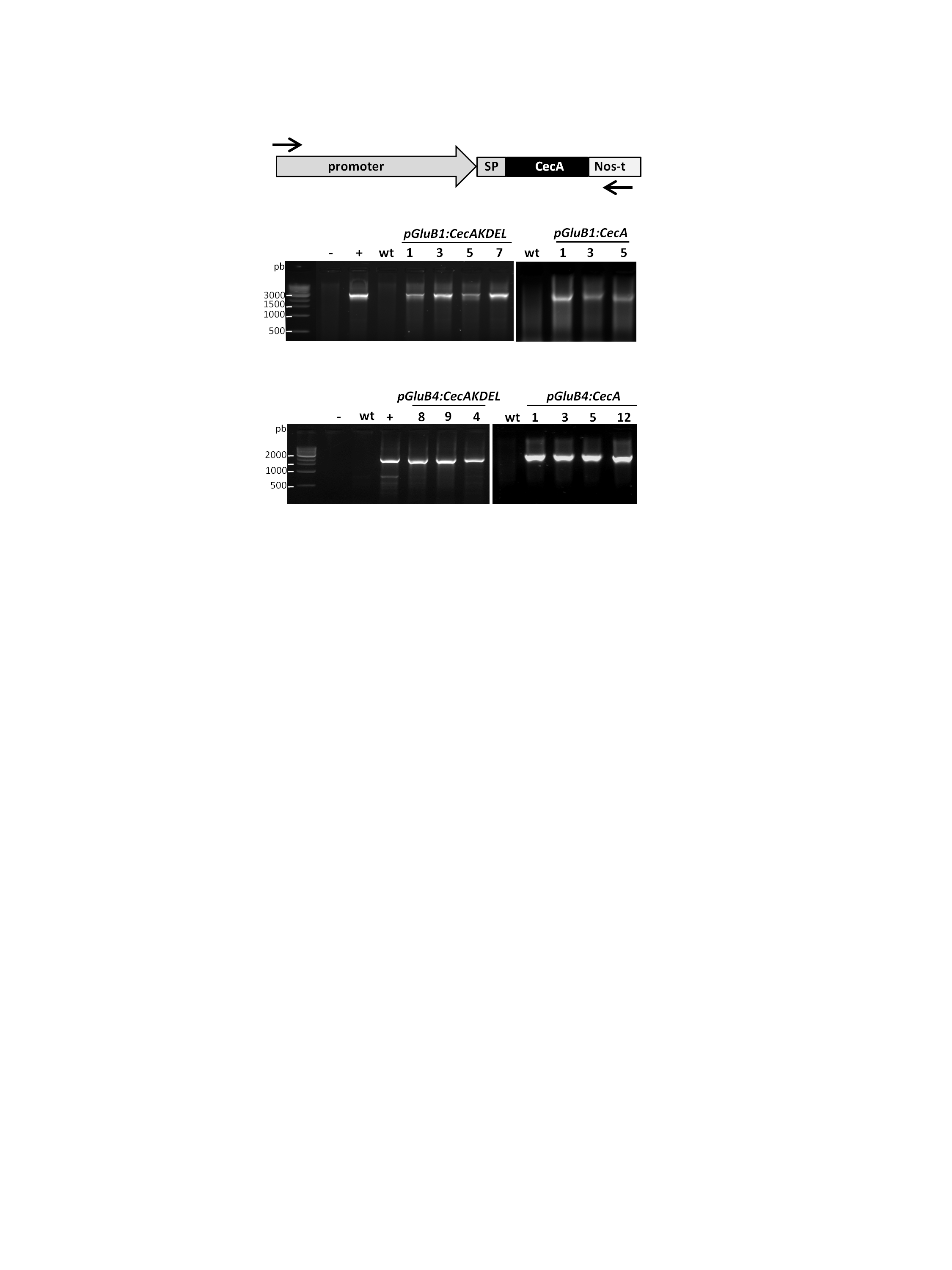

Supplement: Additional file 1 — Confirmation of the transgene insertion in the genome of transgenic rice plants. PCR analysis on genomic DNA purified from leaves of wild-type (wt) or transgenic lines carrying the indicated transgenes. Plasmidic DNA was used as a positive control (+). Arrows indicate the position of the specific oligonucleotides used for PCR amplification. The size of amplified fragments showed full length transgene insertion. [file 1471-2229-14-102-S1.tiff]
