# Supplementary material for: Production of cecropin A antimicrobial peptide in rice seed endosperm
Source: BMC Plant Biol. 2014 Apr 22;14:102. doi: 10.1186/1471-2229-14-102 (PMC4032361; doi:10.1186/1471-2229-14-102)
Supplement: Additional file 2 — Estimated transgene copy number in the transgenic lines by qPCR analysis. [file 1471-2229-14-102-S2.pdf]

Standard curve for the *CecA* gene by qPCR analysis of serial DNA dilution.

Copies of *CecA* gene	Ct values	SD
100000	20.65	0.04
10000	23.83	0.04
1000	27.42	0.15
100	30.75	0.21
10	34.73	0.21

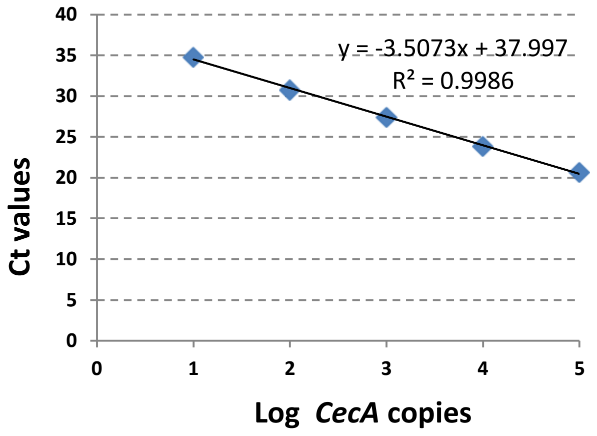

Estimated *CecA* gene copy number in the transgenic lines by qPCR analysis. The value used for calculation was 0.86-0.91 pg of DNA for the rice diploid genome (http://archive.gramene.org/newsletters/rice_genetics/rgn10/vXV32.htm)

Transgene	Event	Ct Value	SD	Copy number 30 ng gDNA	Copy number per genome
None	WT-1	31.69	0.03	62.59	0.00
None	EV-1	31.07	0.22	94.04	0.01
None	EV-2	31.95	0.25	52.89	0.00
pGluB1:CecAKDEL	1	21.75	0.04	42719.64	2.52
	3	21.60	0.05	47140.64	2.78
	5	21.48	0.04	50893.16	3
	7	22.84	0.01	20885.61	1.23
pGluB1:CecA	1	22.21	0.03	31584.39	1.86
	3	23.92	0.03	10300.74	0.61
	5	21.85	0.08	40005.12	2.36
pGluB4:CecAKDEL	8	22.04	0.07	35313.67	2.08
	9	22.10	0.08	34024.06	2.01
	4	21.13	0.05	64320.90	3.79
pGluB4:CecA	1	22.61	0.06	24236.74	1.43
	3	23.64	0.03	12379.47	0.73
	5	23.09	0.03	17724.23	1.05
	12	19.97	0.01	137148.18	8.09
pUbi:CecAKDEL	7	22.08	0.03	34398.39	2.03
pUbi:CecA	2	23.16	0.08	16965.21	1.00
