# Supplementary material for: Production of cecropin A antimicrobial peptide in rice seed endosperm
Source: BMC Plant Biol. 2014 Apr 22;14:102. doi: 10.1186/1471-2229-14-102 (PMC4032361; doi:10.1186/1471-2229-14-102)
Supplement: Additional file 3 — List of primers used in the study. Underlined are the restriction sites used for cloning purposes. [file 1471-2229-14-102-S3.docx]

| Name | Sequence | Restriction site |
| --- | --- | --- |
| promGluB1fwd | 5'GG**GGTACC**TCTAGACAGATTCTTGCTACCAA3' | *Kpn*I |
| promGluB1rev | 5'CC**GAGCTC GGATCC**ATGGCATAGGAGAAGAA3´ | *Sac*I, *Bam*HI |
| promGluB4fwd | 5'GG**GGTACC**TACAGGGTTCCTTGCGTGAAGAA3' | *Kpn*I |
| promGluB4rev | 5'CC**GAGCTC GGATCC**ATGGCATAGGAGAAGAA3' | *Sac*I, *Bam*HI |
| termNosfwd | 5´ GG**GGATCC**CGGGATCGTTCAAACATTTGGCAA3´ | *Bam*HI |
| 1termNosrev | 5'CC**GAGCTC**GTTTGACAGCTTATCATCGGATCTA3' | *Sac*I |
| 2termNosrev | 5'CC**GGTACC**GTTT GACAGCTTATCATCGGATCTA3' | *Kpn*I |
| CecAfwd | 5’GG**GGATCC**ATGGCCAAGTGGAAGCTTTTCAAGAAGAT3’ | *Bam*HI |
| CecArev | 5'CC**GGATCC**ATTATCACTTGGCGAT TTGGTTGGCTT3' | *Bam*HI |
| CecAKDELrev | 5'CC**GGATCC**ATTATCAGAGCTCGTCCTTCTTGGCGATTTGGTTGGCTT3' | *Bam*HI |
| CecA_qPCR_fwd | 5´TCAAGAAGATCGAGAAGGTCGG3´ |  |
| CecA_qPCR_rev | 5´CCTTGATGATGCCGTCGC3´ |  |
